# 1-(4-Chloro­phenyl­sulfon­yl)-5-(4-fluoro­phen­yl)-5-methyl­imidazolidine-2,4-dione

**DOI:** 10.1107/S160053680901037X

**Published:** 2009-03-25

**Authors:** Abid Hussain, Shahid Hameed, Helen Stoeckli-Evans

**Affiliations:** a Department of Chemistry, Quaid-i-Azam University, Islamabad 45320, Pakistan; b Institute of Physics, University of Neuchâtel, rue Emile-Argand 11, CH-2009 Neuchâtel, Switzerland

## Abstract

The title compound, C_16_H_12_ClFN_2_O_4_S, crystallizes with two independent mol­ecules (*A* and *B*) in the asymmetric unit. The two mol­ecules are U-shaped with similar geometries and conformations. The mean planes through the benzene rings are inclined to one another by 6.07 (8)° in mol­ecule *A* and 8.67 (8)° in mol­ecule *B*. They are separated with a centroid–centroid distance of 3.9096 (10) Å in mol­ecule *A* and 3.9118 (10) Å in mol­ecule *B*. Mol­ecules *A* and *B* lie adjacent to one another, with a centroid–centroid distance of 3.7592 (10) Å between the fluoro­phenyl ring of mol­ecule *A* and the chloro­phenyl­sulfonyl ring of mol­ecule *B* and with a dihedral angle of 5.75 (8)° between the ring planes. In the crystal structure, *A* and *B* mol­ecules are linked by N—H⋯O hydrogen bonds, forming centrosymmetric dimers. These dimers stack along the [110] direction and are linked by C—H⋯O and C—H⋯F inter­actions. There are also some short halide⋯halide contacts [Cl⋯F = 3.0499 (14) and 3.1224 (13) Å, and F⋯F = 3.0612 (17) Å].

## Related literature

For the biological activity of imidazolidine-2,4-diones, see: Muccioli *et al.* (2006 ▶); Flosi *et al.* (2006 ▶). For the biological activity of sulfonyl derivatives of imidazolidine-2,4-diones, see: Kato, Nakayama, Mizota *et al.* (1991 ▶); Kato, Nakayama, Ohta *et al.* (1991 ▶); Ahmad *et al.* (2000 ▶, 2002 ▶); Kashif, Ahmad & Hameed (2008 ▶). For the crystal structure of 5-(4-fluoro­phen­yl)-5-methyl­imidazolidine-2,4-dione, see: Kashif, Hussain *et al.* (2008 ▶).
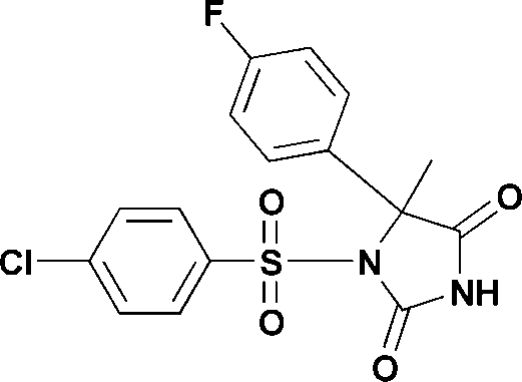


## Experimental

### 

#### Crystal data

C_16_H_12_ClFN_2_O_4_S
*M* *_r_* = 382.79
Triclinic, 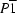

*a* = 9.6959 (7) Å
*b* = 10.0066 (7) Å
*c* = 16.6269 (13) Å
α = 92.098 (6)°
β = 93.630 (6)°
γ = 103.056 (6)°
*V* = 1566.2 (2) Å^3^
*Z* = 4
Mo *K*α radiation
μ = 0.41 mm^−1^
*T* = 173 K
0.45 × 0.40 × 0.24 mm

#### Data collection

Stoe IPDS-2 diffractometer
Absorption correction: none
30542 measured reflections
8438 independent reflections
5836 reflections with *I* > 2σ(*I*)
*R*_int_ = 0.042

#### Refinement

*R*[*F* ^2^ > 2σ(*F* ^2^)] = 0.037
*wR*(*F* ^2^) = 0.095
*S* = 0.89
8438 reflections
462 parameters
H atoms treated by a mixture of independent and constrained refinement
Δρ_max_ = 0.49 e Å^−3^
Δρ_min_ = −0.64 e Å^−3^

### 

Data collection: *X-AREA* (Stoe & Cie, 2006 ▶); cell refinement: *X-AREA*; data reduction: *X-RED32* (Stoe & Cie, 2006 ▶); program(s) used to solve structure: *SHELXS97* (Sheldrick, 2008 ▶); program(s) used to refine structure: *SHELXL97* (Sheldrick, 2008 ▶); molecular graphics: *PLATON* (Spek, 2009 ▶); software used to prepare material for publication: *SHELXL97*.

## Supplementary Material

Crystal structure: contains datablocks I, global. DOI: 10.1107/S160053680901037X/nc2138sup1.cif

Structure factors: contains datablocks I. DOI: 10.1107/S160053680901037X/nc2138Isup2.hkl

Additional supplementary materials:  crystallographic information; 3D view; checkCIF report

## Figures and Tables

**Table 1:** Hydrogen-bond geometry (Å, °)

*D*—H⋯*A*	*D*—H	H⋯*A*	*D*⋯*A*	*D*—H⋯*A*
N2—H2*N*⋯O5^i^	0.84 (2)	2.23 (2)	2.9383 (17)	143.0 (18)
N4—H4*N*⋯O1^i^	0.875 (19)	2.103 (19)	2.8595 (17)	144.4 (17)
C6—H6⋯O6^ii^	0.95	2.53	3.339 (2)	143
C16—H16⋯O7^iii^	0.95	2.38	3.2330 (18)	149
C26—H26*C*⋯O4^iv^	0.98	2.41	3.358 (2)	162
C32—H32⋯O3^v^	0.95	2.38	3.2715 (19)	155
C3—H3⋯F1^vi^	0.95	2.76	3.308 (2)	118
C29—H29⋯F1^vii^	0.95	2.66	3.3715 (19)	133
C13—H13⋯F2^vii^	0.95	2.75	3.4627 (19)	133
C19—H19⋯F2^vii^	0.95	2.69	3.223 (2)	116

Symmetry codes: (i) 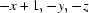; (ii) 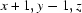; (iii) 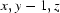; (iv) 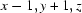; (v) 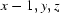; (vi) 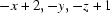; (vii) 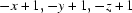.

**Fig. 1.**
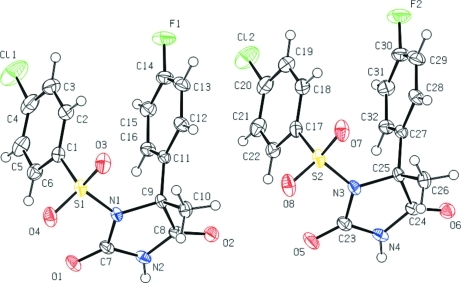
A view of molecular structure of the two independent molecules (A-left & B-right) of the title compound. The displacement ellipsoids are drawn at the 50% probability level.

**Fig. 2.**
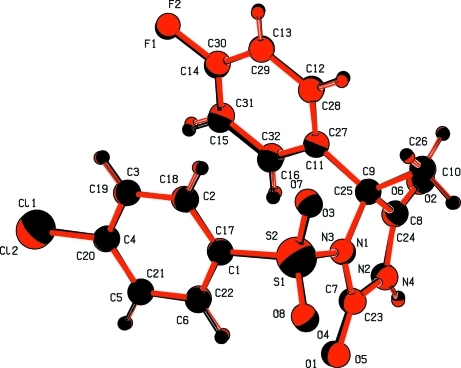
An auto-fit view (Spek, 2009) of the two independent molecules (A black; B red) in the title compound.

**Fig. 3.**
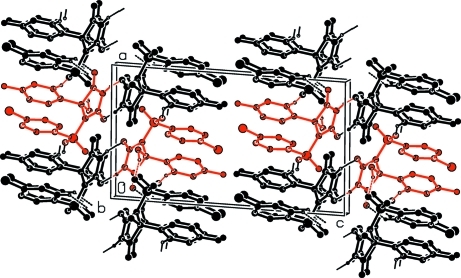
A view along the b axis showing the crystal packing in the title compound: Molecule A is black; Molecule B is red [The N—H···O and C—H···O hydrogen bonds are shown as dashed lines; H-atoms not involved in hydrogen bonding have been removed for clarity; see Table 1 for details].

## supplementary crystallographic information

### Comment

Immidazolidine-2,4-diones are a valuable class of cyclic urea derivatives exhibiting a broad spectrum of activities ranging from fatty acid amide hydrolase inhibitors (Muccioli *et al.*, 2006) to HIV protease inhibitors (Flosi *et al.*, 2006). Their sulfonyl derivatives not only possess strong inhibitory activity against aldose reductase (Kato, Nakayama, Mizota *et al.*, 1991) but also address diabetic complications such as neuropathy and cataract formation (Kato, Nakayama, Ohta, *et al.*, 1991). Hypoglycemic and aldose reductase inhibitory assay of this novel class of compounds has been reported from this laboratory (Ahmad *et al.*, 2000; Ahmad *et al.*, 2002; Kashif, Ahmad & Hameed, 2008). The crystal structure of 5-(4-Fluorophenyl)-5-methylimidazolidine-2,4-dione (Kashif, Hussain *et al.*, 2008) has been reported previously. The title compound was synthesized by sulfonylation of 5-(4-Fluorophenyl)-5-methylimidazolidine-2,4-dione, to access its hypoglycemic activity.

The title compound crystallizes with two independent molecues (A and B) in the asymmetric unit. It is composed of a methylimidazolidine-2,4-dione moiety substituted with a *p*-fluorophenyl group and a *p*-chlorophenylsulfonyl group. Both molecules are U-shaped with a similar geometry and conformation, as can be seen from the Auto-Fit diagram (Fig. 2: Weighted and Unit Weight RMS-Fit = 0.075, 0.069 Å, respectively, for 25 non-H atoms; Spek, 2009). The phenyl ring mean planes are inclined to one another by 6.07 (8)° in molecule A and 8.67 (8)° in molecule B. They are separated with a centroid-to-centroid distance of 3.9096 (10) Å in molecule A, and 3.9118 (10) Å in molecule B. Molecules A and B lie adjacent to one another with a centroid-to-centroid distance of 3.7591 (10) Å between the fluorophenyl ring of molecule A and the chlorophenylsulfonyl ring of molecule B (Fig. 1).

In the crystal structure of the title compound the A and B molecules are linked by N—H···O hydrogen bonds to form a dimer-like arrangement (Fig. 3 and Table 1). These dimers stack along the direction [110] and are linked by C—H···O interactions. The stacks are also linked by C—H···F interactions, and there are some short halide···halide contacts: 3.0499 (14) Å for Cl1···F2^i^ and 3.1224 (13) Å,  for Cl2···F1^i^ [symmetry operation (i) -x+1, -y, -z+1]) and 3.0612 (17) Å for F1···F2^ii^ [symmetry operation (ii) -x+1, -y+1, -z+1].

### Experimental

5-(4-Fluorophenyl)-5-methylimidazolidine-2,4-dione (4.8 mmol) in CH_2_Cl_2_ (20 ml) as stirred with triethyl amine (4.8 mmol) and catalytic amounts of DMAP. 4-Chlorobenzene sulfonyl chloride (5.8 mmol) in CH_2_Cl_2_ (10 ml) was added drop wise and the reaction mixture was stirred at rt until complete (controled by TLC). The reaction mixture was diluted with 1 N HCl (20 ml) and extracted with CH_2_Cl_2_ (3 × 25 ml). The organic layer was dried over anhydrous sodium sulfate and concentrated under reduced pressure. Crystallization of the residue in ethyl acetate afforded the title compound as colourless plate-like crystals, suitable for X-ray analysis.

### Refinement

The NH H-atoms were located in difference Fourier maps and freely refined: N—H = 0.84 (2) - 0.875 (19) Å. The other H-atoms were included in calculated positions and treated as riding atoms: C—H = 0.995 - 0.98 Å, with U_iso_(H) = k × U_eq_(parent C-atom), where k = 1.2 (aromatic H) and 1.5(methyl H).

### Crystal data

**Table:**

C_16_H_12_ClFN_2_O_4_S	*Z* = 4
*M**_r_* = 382.79	*F*(000) = 784
Triclinic, *P*1	*D*_x_ = 1.623 Mg m^−^^3^
Hall symbol: -P 1	Mo *K*α radiation, λ = 0.71073 Å
*a* = 9.6959 (7) Å	Cell parameters from 20500 reflections
*b* = 10.0066 (7) Å	θ = 2.1–29.5°
*c* = 16.6269 (13) Å	µ = 0.41 mm^−^^1^
α = 92.098 (6)°	*T* = 173 K
β = 93.630 (6)°	Plate, colourless
γ = 103.056 (6)°	0.45 × 0.40 × 0.24 mm
*V* = 1566.2 (2) Å^3^	

### Data collection

**Table:**

Stoe IPDS-2 diffractometer	5836 reflections with *I* > 2σ(*I*)
Radiation source: fine-focus sealed tube	*R*_int_ = 0.042
graphite	θ_max_ = 29.3°, θ_min_ = 2.1°
φ and ω scans	*h* = −13→12
30542 measured reflections	*k* = −13→13
8438 independent reflections	*l* = −22→22

### Refinement

**Table:**

Refinement on *F*^2^	Secondary atom site location: difference Fourier map
Least-squares matrix: full	Hydrogen site location: inferred from neighbouring sites
*R*[*F*^2^ > 2σ(*F*^2^)] = 0.037	H atoms treated by a mixture of independent and constrained refinement
*wR*(*F*^2^) = 0.095	*w* = 1/[σ^2^(*F*_o_^2^) + (0.0594*P*)^2^] where *P* = (*F*_o_^2^ + 2*F*_c_^2^)/3
*S* = 0.89	(Δ/σ)_max_ = 0.001
8438 reflections	Δρ_max_ = 0.49 e Å^−^^3^
462 parameters	Δρ_min_ = −0.64 e Å^−^^3^
0 restraints	Extinction correction: *SHELXL97* (Sheldrick, 2008), Fc^*^=kFc[1+0.001xFc^2^λ^3^/sin(2θ)]^-1/4^
Primary atom site location: structure-invariant direct methods	Extinction coefficient: 0.0070 (7)

### Special details

**Table:**

Geometry. Bond distances, angles *etc*. have been calculated using the rounded fractional coordinates. All su's are estimated from the variances of the (full) variance-covariance matrix. The cell e.s.d.'s are taken into account in the estimation of distances, angles and torsion angles
Refinement. Refinement of *F*^2^ against ALL reflections. The weighted *R*-factor *wR* and goodness of fit *S* are based on *F*^2^, conventional *R*-factors *R* are based on *F*, with *F* set to zero for negative *F*^2^. The threshold expression of *F*^2^ > σ(*F*^2^) is used only for calculating *R*-factors(gt) *etc*. and is not relevant to the choice of reflections for refinement. *R*-factors based on *F*^2^ are statistically about twice as large as those based on *F*, and *R*- factors based on ALL data will be even larger.

### Fractional atomic coordinates and isotropic or equivalent isotropic displacement parameters (Å^2^)

**Table:**

	*x*	*y*	*z*	*U*_iso_*/*U*_eq_	
Cl1	0.91764 (7)	−0.33306 (8)	0.44481 (3)	0.0678 (2)	
S1	1.07493 (4)	0.03707 (4)	0.15675 (2)	0.0216 (1)	
F1	0.74943 (12)	0.11810 (12)	0.46455 (6)	0.0435 (4)	
O1	0.88878 (13)	−0.18444 (11)	0.04068 (7)	0.0292 (3)	
O2	0.57120 (12)	0.08199 (11)	0.07970 (6)	0.0266 (3)	
O3	1.12161 (12)	0.17630 (11)	0.18656 (8)	0.0317 (3)	
O4	1.15471 (12)	−0.02016 (12)	0.10155 (7)	0.0306 (3)	
N1	0.91377 (13)	0.02536 (12)	0.11173 (7)	0.0188 (3)	
N2	0.70525 (14)	−0.07265 (13)	0.04699 (7)	0.0217 (4)	
C1	1.04515 (16)	−0.06802 (16)	0.23943 (9)	0.0239 (4)	
C2	1.06304 (19)	−0.00804 (19)	0.31679 (10)	0.0324 (5)	
C3	1.0280 (2)	−0.0905 (2)	0.38108 (10)	0.0412 (6)	
C4	0.9731 (2)	−0.2300 (2)	0.36571 (11)	0.0399 (6)	
C5	0.95939 (19)	−0.29070 (19)	0.28916 (11)	0.0350 (5)	
C6	0.99744 (17)	−0.20954 (16)	0.22461 (10)	0.0271 (5)	
C7	0.84130 (17)	−0.08949 (14)	0.06303 (8)	0.0211 (4)	
C8	0.68087 (16)	0.04368 (14)	0.08337 (8)	0.0192 (4)	
C9	0.81941 (15)	0.11962 (13)	0.13079 (8)	0.0175 (4)	
C10	0.86819 (16)	0.25816 (14)	0.09386 (9)	0.0227 (4)	
C11	0.79666 (15)	0.12483 (14)	0.22069 (8)	0.0180 (4)	
C12	0.84186 (16)	0.24448 (15)	0.26969 (9)	0.0230 (4)	
C13	0.82563 (18)	0.24327 (17)	0.35230 (9)	0.0287 (5)	
C14	0.76299 (18)	0.12121 (18)	0.38395 (9)	0.0282 (5)	
C15	0.71357 (17)	0.00078 (16)	0.33735 (9)	0.0264 (4)	
C16	0.73163 (16)	0.00364 (15)	0.25529 (8)	0.0221 (4)	
Cl2	0.40511 (6)	0.12790 (6)	0.43991 (3)	0.0533 (2)	
S2	0.58277 (4)	0.54282 (4)	0.17383 (2)	0.0238 (1)	
F2	0.23250 (13)	0.58675 (13)	0.47233 (6)	0.0462 (4)	
O5	0.41852 (14)	0.33765 (12)	0.04019 (7)	0.0323 (4)	
O6	0.08075 (12)	0.57818 (11)	0.08571 (6)	0.0268 (3)	
O7	0.62142 (12)	0.67806 (11)	0.21087 (8)	0.0329 (4)	
O8	0.67356 (13)	0.49688 (12)	0.12089 (8)	0.0349 (4)	
N3	0.42774 (14)	0.53320 (12)	0.12274 (7)	0.0207 (4)	
N4	0.22860 (15)	0.43999 (13)	0.04734 (7)	0.0245 (4)	
C17	0.54287 (17)	0.42502 (15)	0.25006 (9)	0.0246 (4)	
C18	0.55335 (19)	0.47308 (18)	0.32980 (10)	0.0314 (5)	
C19	0.5129 (2)	0.3808 (2)	0.38883 (10)	0.0366 (6)	
C20	0.46166 (19)	0.24306 (19)	0.36642 (10)	0.0340 (5)	
C21	0.45376 (18)	0.19449 (17)	0.28735 (10)	0.0313 (5)	
C22	0.49622 (17)	0.28577 (15)	0.22782 (9)	0.0258 (4)	
C23	0.36440 (17)	0.42562 (15)	0.06618 (8)	0.0234 (4)	
C24	0.19359 (16)	0.54614 (14)	0.08942 (8)	0.0203 (4)	
C25	0.32640 (15)	0.62082 (13)	0.14231 (8)	0.0181 (4)	
C26	0.37432 (17)	0.76406 (14)	0.11053 (9)	0.0235 (4)	
C27	0.29616 (15)	0.61719 (14)	0.23119 (8)	0.0188 (4)	
C28	0.34294 (17)	0.73075 (15)	0.28478 (9)	0.0250 (4)	
C29	0.32074 (18)	0.72166 (17)	0.36611 (10)	0.0312 (5)	
C30	0.25095 (18)	0.59752 (18)	0.39255 (9)	0.0300 (5)	
C31	0.19982 (18)	0.48331 (17)	0.34161 (9)	0.0280 (5)	
C32	0.22340 (16)	0.49359 (15)	0.26027 (9)	0.0226 (4)	
H2	1.09880	0.08820	0.32580	0.0390*	
H2N	0.646 (2)	−0.124 (2)	0.0142 (12)	0.037 (5)*	
H3	1.04140	−0.05190	0.43480	0.0490*	
H5	0.92420	−0.38710	0.28060	0.0420*	
H6	0.99110	−0.24960	0.17140	0.0330*	
H10A	0.88320	0.24360	0.03680	0.0340*	
H10B	0.95730	0.30860	0.12250	0.0340*	
H10C	0.79550	0.31140	0.09860	0.0340*	
H12	0.88430	0.32790	0.24650	0.0280*	
H13	0.85700	0.32470	0.38590	0.0340*	
H15	0.66860	−0.08150	0.36080	0.0320*	
H16	0.69920	−0.07820	0.22210	0.0260*	
H4N	0.170 (2)	0.3855 (19)	0.0117 (11)	0.031 (5)*	
H18	0.58780	0.56820	0.34390	0.0380*	
H19	0.52030	0.41170	0.44400	0.0440*	
H21	0.41950	0.09920	0.27360	0.0370*	
H22	0.49350	0.25380	0.17310	0.0310*	
H26A	0.38990	0.75590	0.05310	0.0350*	
H26B	0.46290	0.81250	0.14050	0.0350*	
H26C	0.30080	0.81560	0.11770	0.0350*	
H28	0.39080	0.81570	0.26540	0.0300*	
H29	0.35290	0.79930	0.40270	0.0370*	
H31	0.14980	0.39970	0.36150	0.0340*	
H32	0.18970	0.41570	0.22400	0.0270*	

### Atomic displacement parameters (Å^2^)

**Table:**

	*U*^11^	*U*^22^	*U*^33^	*U*^12^	*U*^13^	*U*^23^
Cl1	0.0535 (3)	0.1057 (5)	0.0463 (3)	0.0147 (3)	0.0103 (2)	0.0444 (3)
S1	0.0190 (2)	0.0171 (2)	0.0295 (2)	0.0054 (1)	0.0028 (1)	0.0019 (1)
F1	0.0520 (7)	0.0584 (7)	0.0186 (4)	0.0092 (6)	0.0052 (4)	−0.0020 (4)
O1	0.0364 (7)	0.0210 (5)	0.0314 (6)	0.0107 (5)	0.0030 (5)	−0.0080 (4)
O2	0.0221 (6)	0.0302 (6)	0.0276 (5)	0.0082 (5)	−0.0011 (4)	−0.0042 (4)
O3	0.0229 (6)	0.0182 (5)	0.0510 (7)	0.0013 (4)	−0.0054 (5)	−0.0014 (5)
O4	0.0285 (6)	0.0317 (6)	0.0378 (6)	0.0155 (5)	0.0141 (5)	0.0092 (5)
N1	0.0203 (6)	0.0147 (5)	0.0224 (6)	0.0064 (5)	0.0025 (5)	−0.0006 (4)
N2	0.0227 (7)	0.0201 (6)	0.0204 (6)	0.0017 (5)	0.0021 (5)	−0.0065 (5)
C1	0.0226 (8)	0.0254 (8)	0.0254 (7)	0.0096 (6)	0.0004 (6)	0.0015 (6)
C2	0.0339 (9)	0.0370 (9)	0.0283 (8)	0.0156 (7)	−0.0056 (7)	−0.0050 (7)
C3	0.0420 (11)	0.0633 (13)	0.0245 (8)	0.0260 (10)	0.0002 (7)	0.0011 (8)
C4	0.0297 (9)	0.0606 (13)	0.0338 (9)	0.0160 (9)	0.0046 (7)	0.0213 (8)
C5	0.0292 (9)	0.0355 (9)	0.0400 (9)	0.0057 (7)	−0.0013 (7)	0.0139 (7)
C6	0.0277 (8)	0.0244 (8)	0.0290 (8)	0.0057 (6)	−0.0006 (6)	0.0042 (6)
C7	0.0283 (8)	0.0156 (6)	0.0192 (6)	0.0045 (6)	0.0047 (5)	−0.0015 (5)
C8	0.0220 (7)	0.0182 (6)	0.0164 (6)	0.0021 (5)	0.0027 (5)	0.0005 (5)
C9	0.0191 (7)	0.0127 (6)	0.0214 (6)	0.0048 (5)	0.0029 (5)	−0.0004 (5)
C10	0.0249 (8)	0.0166 (7)	0.0279 (7)	0.0057 (6)	0.0049 (6)	0.0057 (5)
C11	0.0185 (7)	0.0152 (6)	0.0200 (6)	0.0038 (5)	0.0013 (5)	−0.0012 (5)
C12	0.0232 (8)	0.0177 (7)	0.0263 (7)	0.0016 (6)	0.0030 (6)	−0.0031 (5)
C13	0.0297 (9)	0.0279 (8)	0.0269 (7)	0.0054 (7)	0.0000 (6)	−0.0102 (6)
C14	0.0285 (8)	0.0390 (9)	0.0179 (7)	0.0097 (7)	0.0028 (6)	−0.0012 (6)
C15	0.0287 (8)	0.0264 (8)	0.0242 (7)	0.0054 (6)	0.0041 (6)	0.0048 (6)
C16	0.0249 (8)	0.0180 (7)	0.0223 (7)	0.0029 (6)	0.0018 (6)	0.0005 (5)
Cl2	0.0499 (3)	0.0667 (4)	0.0426 (3)	0.0081 (3)	0.0044 (2)	0.0272 (2)
S2	0.0205 (2)	0.0163 (2)	0.0358 (2)	0.0056 (1)	0.0049 (2)	0.0018 (1)
F2	0.0560 (7)	0.0628 (7)	0.0196 (5)	0.0125 (6)	0.0054 (5)	0.0011 (4)
O5	0.0425 (7)	0.0245 (6)	0.0328 (6)	0.0144 (5)	0.0061 (5)	−0.0079 (4)
O6	0.0237 (6)	0.0278 (6)	0.0280 (5)	0.0056 (5)	0.0004 (4)	−0.0041 (4)
O7	0.0245 (6)	0.0162 (5)	0.0560 (8)	0.0032 (4)	−0.0033 (5)	−0.0025 (5)
O8	0.0296 (6)	0.0311 (6)	0.0500 (7)	0.0148 (5)	0.0166 (5)	0.0097 (5)
N3	0.0250 (7)	0.0154 (6)	0.0231 (6)	0.0069 (5)	0.0047 (5)	−0.0009 (4)
N4	0.0304 (7)	0.0214 (6)	0.0206 (6)	0.0045 (5)	0.0023 (5)	−0.0065 (5)
C17	0.0227 (8)	0.0210 (7)	0.0316 (8)	0.0090 (6)	−0.0001 (6)	0.0003 (6)
C18	0.0339 (9)	0.0307 (9)	0.0316 (8)	0.0146 (7)	−0.0055 (7)	−0.0052 (6)
C19	0.0395 (10)	0.0476 (11)	0.0269 (8)	0.0208 (8)	−0.0030 (7)	−0.0006 (7)
C20	0.0297 (9)	0.0434 (10)	0.0318 (8)	0.0131 (8)	0.0017 (7)	0.0126 (7)
C21	0.0288 (9)	0.0251 (8)	0.0383 (9)	0.0037 (7)	−0.0029 (7)	0.0061 (7)
C22	0.0290 (8)	0.0207 (7)	0.0277 (7)	0.0066 (6)	−0.0004 (6)	0.0007 (6)
C23	0.0304 (8)	0.0185 (7)	0.0218 (7)	0.0058 (6)	0.0054 (6)	−0.0002 (5)
C24	0.0253 (8)	0.0166 (6)	0.0183 (6)	0.0024 (6)	0.0042 (5)	0.0014 (5)
C25	0.0196 (7)	0.0128 (6)	0.0226 (6)	0.0045 (5)	0.0040 (5)	0.0005 (5)
C26	0.0241 (8)	0.0142 (6)	0.0326 (8)	0.0040 (6)	0.0053 (6)	0.0048 (5)
C27	0.0196 (7)	0.0157 (6)	0.0207 (6)	0.0037 (5)	0.0015 (5)	−0.0016 (5)
C28	0.0261 (8)	0.0176 (7)	0.0296 (7)	0.0022 (6)	0.0030 (6)	−0.0053 (5)
C29	0.0330 (9)	0.0314 (8)	0.0273 (8)	0.0061 (7)	−0.0001 (7)	−0.0116 (6)
C30	0.0314 (9)	0.0418 (10)	0.0180 (7)	0.0108 (7)	0.0026 (6)	−0.0008 (6)
C31	0.0297 (9)	0.0276 (8)	0.0259 (7)	0.0036 (7)	0.0035 (6)	0.0055 (6)
C32	0.0247 (8)	0.0181 (7)	0.0231 (7)	0.0017 (6)	0.0009 (6)	−0.0004 (5)

### Geometric parameters (Å, °)

**Table:**

Cl1—C4	1.738 (2)	C14—C15	1.381 (2)
Cl2—C20	1.7390 (18)	C15—C16	1.387 (2)
S1—O4	1.4214 (12)	C2—H2	0.9500
S1—N1	1.6669 (13)	C3—H3	0.9500
S1—C1	1.7601 (16)	C5—H5	0.9500
S1—O3	1.4240 (12)	C6—H6	0.9500
S2—O7	1.4254 (12)	C10—H10A	0.9800
S2—O8	1.4186 (14)	C10—H10B	0.9800
S2—C17	1.7638 (15)	C10—H10C	0.9800
S2—N3	1.6605 (14)	C12—H12	0.9500
F1—C14	1.3556 (18)	C13—H13	0.9500
F2—C30	1.3546 (18)	C15—H15	0.9500
O1—C7	1.2026 (19)	C16—H16	0.9500
O2—C8	1.208 (2)	C17—C22	1.393 (2)
O5—C23	1.203 (2)	C17—C18	1.383 (2)
O6—C24	1.206 (2)	C18—C19	1.386 (2)
N1—C7	1.4018 (18)	C19—C20	1.384 (3)
N1—C9	1.4939 (19)	C20—C21	1.376 (2)
N2—C7	1.378 (2)	C21—C22	1.390 (2)
N2—C8	1.3666 (19)	C24—C25	1.537 (2)
N2—H2N	0.84 (2)	C25—C26	1.5281 (19)
N3—C23	1.4052 (18)	C25—C27	1.5249 (19)
N3—C25	1.4996 (19)	C27—C32	1.398 (2)
N4—C23	1.375 (2)	C27—C28	1.390 (2)
N4—C24	1.3681 (19)	C28—C29	1.386 (2)
N4—H4N	0.875 (19)	C29—C30	1.376 (2)
C1—C6	1.394 (2)	C30—C31	1.375 (2)
C1—C2	1.383 (2)	C31—C32	1.390 (2)
C2—C3	1.387 (2)	C18—H18	0.9500
C3—C4	1.385 (3)	C19—H19	0.9500
C4—C5	1.376 (3)	C21—H21	0.9500
C5—C6	1.388 (2)	C22—H22	0.9500
C8—C9	1.538 (2)	C26—H26A	0.9800
C9—C10	1.5250 (19)	C26—H26B	0.9800
C9—C11	1.5254 (19)	C26—H26C	0.9800
C11—C16	1.397 (2)	C28—H28	0.9500
C11—C12	1.391 (2)	C29—H29	0.9500
C12—C13	1.393 (2)	C31—H31	0.9500
C13—C14	1.375 (2)	C32—H32	0.9500
			
O3—S1—O4	120.54 (7)	H10B—C10—H10C	109.00
O3—S1—N1	105.32 (7)	C9—C10—H10A	109.00
O3—S1—C1	108.55 (8)	C9—C10—H10C	109.00
O4—S1—N1	107.44 (7)	H10A—C10—H10B	109.00
O4—S1—C1	109.31 (7)	C13—C12—H12	120.00
N1—S1—C1	104.49 (7)	C11—C12—H12	120.00
O8—S2—N3	107.85 (7)	C14—C13—H13	121.00
O8—S2—C17	109.35 (8)	C12—C13—H13	121.00
N3—S2—C17	104.29 (7)	C16—C15—H15	121.00
O7—S2—C17	108.38 (7)	C14—C15—H15	121.00
O7—S2—O8	120.43 (7)	C15—C16—H16	119.00
O7—S2—N3	105.34 (7)	C11—C16—H16	119.00
C7—N1—C9	111.78 (12)	S2—C17—C18	119.53 (12)
S1—N1—C9	124.37 (9)	C18—C17—C22	121.65 (14)
S1—N1—C7	122.81 (11)	S2—C17—C22	118.78 (11)
C7—N2—C8	113.69 (12)	C17—C18—C19	119.20 (16)
C8—N2—H2N	122.9 (14)	C18—C19—C20	119.16 (16)
C7—N2—H2N	123.1 (14)	Cl2—C20—C21	118.94 (14)
S2—N3—C25	124.18 (9)	Cl2—C20—C19	119.28 (13)
S2—N3—C23	123.49 (11)	C19—C20—C21	121.79 (16)
C23—N3—C25	111.33 (12)	C20—C21—C22	119.53 (15)
C23—N4—C24	114.09 (12)	C17—C22—C21	118.62 (14)
C24—N4—H4N	123.3 (13)	N3—C23—N4	106.55 (13)
C23—N4—H4N	122.6 (13)	O5—C23—N3	126.46 (15)
S1—C1—C6	118.72 (12)	O5—C23—N4	126.98 (14)
C2—C1—C6	121.81 (15)	O6—C24—N4	127.18 (14)
S1—C1—C2	119.40 (13)	O6—C24—C25	125.42 (13)
C1—C2—C3	119.02 (17)	N4—C24—C25	107.39 (13)
C2—C3—C4	118.98 (16)	N3—C25—C24	100.55 (10)
Cl1—C4—C3	119.61 (14)	C24—C25—C26	107.20 (11)
Cl1—C4—C5	118.26 (15)	C24—C25—C27	110.25 (12)
C3—C4—C5	122.13 (17)	C26—C25—C27	115.47 (11)
C4—C5—C6	119.27 (17)	N3—C25—C26	110.75 (12)
C1—C6—C5	118.64 (15)	N3—C25—C27	111.50 (11)
O1—C7—N1	126.44 (15)	C28—C27—C32	118.95 (13)
N1—C7—N2	106.47 (12)	C25—C27—C28	122.38 (12)
O1—C7—N2	127.08 (14)	C25—C27—C32	118.63 (12)
N2—C8—C9	107.77 (13)	C27—C28—C29	120.87 (14)
O2—C8—C9	125.18 (13)	C28—C29—C30	118.37 (15)
O2—C8—N2	127.05 (14)	F2—C30—C31	118.41 (15)
C10—C9—C11	115.85 (11)	F2—C30—C29	118.72 (15)
N1—C9—C8	100.25 (10)	C29—C30—C31	122.88 (15)
N1—C9—C10	111.26 (12)	C30—C31—C32	118.17 (15)
C8—C9—C10	107.59 (11)	C27—C32—C31	120.74 (14)
C8—C9—C11	109.55 (12)	C17—C18—H18	120.00
N1—C9—C11	111.12 (11)	C19—C18—H18	120.00
C12—C11—C16	118.98 (13)	C18—C19—H19	120.00
C9—C11—C16	118.47 (12)	C20—C19—H19	120.00
C9—C11—C12	122.52 (12)	C20—C21—H21	120.00
C11—C12—C13	120.71 (14)	C22—C21—H21	120.00
C12—C13—C14	118.31 (14)	C17—C22—H22	121.00
F1—C14—C15	118.21 (15)	C21—C22—H22	121.00
C13—C14—C15	122.95 (14)	C25—C26—H26A	109.00
F1—C14—C13	118.84 (15)	C25—C26—H26B	109.00
C14—C15—C16	117.94 (14)	C25—C26—H26C	109.00
C11—C16—C15	121.08 (13)	H26A—C26—H26B	110.00
C3—C2—H2	120.00	H26A—C26—H26C	109.00
C1—C2—H2	121.00	H26B—C26—H26C	109.00
C2—C3—H3	120.00	C27—C28—H28	120.00
C4—C3—H3	121.00	C29—C28—H28	120.00
C4—C5—H5	120.00	C28—C29—H29	121.00
C6—C5—H5	120.00	C30—C29—H29	121.00
C1—C6—H6	121.00	C30—C31—H31	121.00
C5—C6—H6	121.00	C32—C31—H31	121.00
C9—C10—H10B	109.00	C27—C32—H32	120.00
H10A—C10—H10C	109.00	C31—C32—H32	120.00
			
O3—S1—N1—C7	169.06 (11)	C2—C3—C4—C5	3.6 (3)
O3—S1—N1—C9	−23.58 (13)	Cl1—C4—C5—C6	177.33 (14)
O4—S1—N1—C7	39.41 (13)	C3—C4—C5—C6	−2.1 (3)
O4—S1—N1—C9	−153.24 (11)	C4—C5—C6—C1	−1.6 (3)
C1—S1—N1—C7	−76.64 (12)	O2—C8—C9—C11	63.69 (17)
C1—S1—N1—C9	90.72 (12)	O2—C8—C9—C10	−63.03 (18)
O3—S1—C1—C2	2.56 (16)	N2—C8—C9—C11	−116.37 (12)
O3—S1—C1—C6	179.52 (13)	N2—C8—C9—N1	0.56 (13)
O4—S1—C1—C2	135.82 (14)	N2—C8—C9—C10	116.91 (12)
O4—S1—C1—C6	−47.21 (15)	O2—C8—C9—N1	−179.38 (13)
N1—S1—C1—C2	−109.44 (14)	C8—C9—C11—C12	−133.46 (14)
N1—S1—C1—C6	67.53 (14)	C8—C9—C11—C16	48.46 (17)
O7—S2—N3—C23	168.37 (12)	C10—C9—C11—C12	−11.6 (2)
O7—S2—N3—C25	−24.06 (13)	C10—C9—C11—C16	170.35 (14)
O8—S2—N3—C23	38.58 (13)	N1—C9—C11—C12	116.69 (15)
O8—S2—N3—C25	−153.85 (11)	N1—C9—C11—C16	−61.39 (17)
C17—S2—N3—C23	−77.60 (13)	C9—C11—C12—C13	−176.64 (15)
C17—S2—N3—C25	89.98 (12)	C12—C11—C16—C15	−0.9 (2)
O7—S2—C17—C18	−0.26 (17)	C16—C11—C12—C13	1.4 (2)
O7—S2—C17—C22	177.31 (13)	C9—C11—C16—C15	177.24 (14)
O8—S2—C17—C18	132.76 (15)	C11—C12—C13—C14	−0.5 (2)
O8—S2—C17—C22	−49.67 (16)	C12—C13—C14—C15	−1.1 (3)
N3—S2—C17—C18	−112.11 (15)	C12—C13—C14—F1	178.57 (15)
N3—S2—C17—C22	65.46 (15)	C13—C14—C15—C16	1.6 (3)
S1—N1—C9—C8	−170.14 (9)	F1—C14—C15—C16	−178.08 (15)
S1—N1—C9—C10	76.29 (14)	C14—C15—C16—C11	−0.6 (2)
S1—N1—C9—C11	−54.40 (15)	S2—C17—C18—C19	175.79 (15)
S1—N1—C7—O1	−8.4 (2)	C22—C17—C18—C19	−1.7 (3)
S1—N1—C7—N2	170.79 (9)	S2—C17—C22—C21	−174.77 (13)
C9—N1—C7—O1	−177.21 (14)	C18—C17—C22—C21	2.8 (3)
C9—N1—C7—N2	2.00 (15)	C17—C18—C19—C20	−0.7 (3)
C7—N1—C9—C8	−1.56 (14)	C18—C19—C20—Cl2	−177.81 (15)
C7—N1—C9—C10	−115.13 (13)	C18—C19—C20—C21	2.0 (3)
C7—N1—C9—C11	114.18 (13)	Cl2—C20—C21—C22	178.86 (14)
C7—N2—C8—O2	−179.43 (14)	C19—C20—C21—C22	−1.0 (3)
C7—N2—C8—C9	0.64 (16)	C20—C21—C22—C17	−1.4 (3)
C8—N2—C7—O1	177.57 (14)	O6—C24—C25—N3	178.72 (13)
C8—N2—C7—N1	−1.63 (16)	O6—C24—C25—C26	−65.50 (18)
C25—N3—C23—O5	−178.52 (14)	O6—C24—C25—C27	60.94 (18)
S2—N3—C23—O5	−9.5 (2)	N4—C24—C25—N3	−2.26 (13)
S2—N3—C23—N4	169.79 (10)	N4—C24—C25—C26	113.52 (13)
C23—N3—C25—C27	117.74 (12)	N4—C24—C25—C27	−120.04 (12)
C25—N3—C23—N4	0.80 (15)	N3—C25—C27—C28	111.40 (15)
S2—N3—C25—C24	−168.02 (9)	N3—C25—C27—C32	−66.12 (17)
S2—N3—C25—C26	78.89 (14)	C24—C25—C27—C28	−137.80 (14)
S2—N3—C25—C27	−51.16 (15)	C24—C25—C27—C32	44.67 (18)
C23—N3—C25—C24	0.88 (14)	C26—C25—C27—C28	−16.1 (2)
C23—N3—C25—C26	−112.22 (13)	C26—C25—C27—C32	166.34 (14)
C23—N4—C24—O6	−177.93 (14)	C25—C27—C28—C29	−176.22 (15)
C24—N4—C23—O5	176.83 (14)	C32—C27—C28—C29	1.3 (2)
C24—N4—C23—N3	−2.48 (16)	C25—C27—C32—C31	176.65 (15)
C23—N4—C24—C25	3.07 (16)	C28—C27—C32—C31	−1.0 (2)
S1—C1—C2—C3	174.66 (14)	C27—C28—C29—C30	−0.1 (3)
C2—C1—C6—C5	3.8 (3)	C28—C29—C30—F2	178.10 (16)
C6—C1—C2—C3	−2.2 (3)	C28—C29—C30—C31	−1.4 (3)
S1—C1—C6—C5	−173.13 (13)	F2—C30—C31—C32	−177.78 (15)
C1—C2—C3—C4	−1.5 (3)	C29—C30—C31—C32	1.8 (3)
C2—C3—C4—Cl1	−175.76 (15)	C30—C31—C32—C27	−0.5 (2)

### Hydrogen-bond geometry (Å, °)

**Table:**

*D*—H···*A*	*D*—H	H···*A*	*D*···*A*	*D*—H···*A*
N2—H2N···O5^i^	0.84 (2)	2.23 (2)	2.9383 (17)	143.0 (18)
N4—H4N···O1^i^	0.875 (19)	2.103 (19)	2.8595 (17)	144.4 (17)
C6—H6···O6^ii^	0.95	2.53	3.339 (2)	143
C16—H16···O7^iii^	0.95	2.38	3.2330 (18)	149
C26—H26C···O4^iv^	0.98	2.41	3.358 (2)	162
C32—H32···O3^v^	0.95	2.38	3.2715 (19)	155
C3—H3···F1^vi^	0.95	2.76	3.308 (2)	118
C29—H29···F1^vii^	0.95	2.66	3.3715 (19)	133
C13—H13···F2^vii^	0.95	2.75	3.4627 (19)	133
C19—H19···F2^vii^	0.95	2.69	3.223 (2)	116

Symmetry codes: (i) −*x*+1, −*y*, −*z*; (ii) *x*+1, *y*−1, *z*; (iii) *x*, *y*−1, *z*; (iv) *x*−1, *y*+1, *z*; (v) *x*−1, *y*, *z*; (vi) −*x*+2, −*y*, −*z*+1; (vii) −*x*+1, −*y*+1, −*z*+1.
